# Modified Iron Deposition in Nigrosomes by Pharmacotherapy for the Management of Parkinson’s Disease

**DOI:** 10.3389/fmolb.2022.908298

**Published:** 2022-07-07

**Authors:** Mengdi Wang, Hongxia Wang, Jing Wang, Shujun Lu, Chen Li, Xiaofei Zhong, Nan Wang, Ruli Ge, Qi Zheng, Jinbo Chen, Hongcai Wang

**Affiliations:** ^1^ Department of Neurology, Binzhou Medical University Hospital, Binzhou, China; ^2^ Department of Radiology, Binzhou Medical University Hospital, Binzhou, China; ^3^ Medical Research Center, Binzhou Medical University Hospital, Binzhou, China

**Keywords:** iron accumulation, nigrosome, Parkinson’s disease, levodopa, dorsolateral nigral hyperintensity

## Abstract

**Background:** Increased iron deposition in nigrosome as assessed by susceptibility-weighted imaging (SWI) is involved in the pathogenesis of Parkinson’s disease (PD). This study investigated the effects of antiparkinson drugs on iron deposition in the nigrosome of PD patients.

**Methods:** Based on the retrospective analysis of clinical data, alterations in iron deposition in the substantia nigra were investigated in 51 PD patients across different types of therapies and in nine Parkinson-plus syndrome patients. The Movement Disorder Society revision of the Unified Parkinson’s Disease Rating Scale (MDS-UPDRS) Part Ⅲ/Ⅳ (UPDRS Ⅲ/Ⅳ) was utilized to evaluate motor function and complications. SWI (slice = 0.6 mm) was used to detect iron deposition in the nigrosome and substantia nigra. Nigrosome loss was scored on a 1-point nigrosome visibility scale. Visual assessment of dorsolateral nigral hyperintensity (DNH) was separately performed for each side of the nigrosome with SWI.

**Results:** Increased UPDRS Ⅲ scores were correlated with low nigrosome scores based on correlation analysis at a disease duration of 6–12 months (*r* = −0.8420). The loss of the nigrosome on SWI was clearly inhibited in PD patients with a 3–5-year duration of administration of antiparkinson medications compared with no treatment. Decreased UPDRS Ⅲ scores and increased nigrosome scores were observed in the regular treatment of PD patients with a 6–7-year disease duration. For patients with Parkinson-plus syndromes, such as multiple system atrophy, iron accumulation was apparent in the corpus striatum and substantia nigra compared with that for patients with progressive supranuclear palsy.

**Conclusions:** Early and regular treatment with antiparkinson drugs not only alleviates the chance of PD disability but also prevents the loss of DNH, namely, iron accumulation in the nigrosome.

## Introduction

Parkinson’s disease (PD) is a progressive degenerative disorder characterized by the selective loss of dopaminergic neurons in the substantia nigra pars compacta (SNc) (Kinoshita et al., 2015). Iron-specific accumulation in the SNc but not in other iron-rich areas is a cardinal feature of degenerating regions in a PD brain (Hare and Double, 2016; Billings et al., 2019). Susceptibility-weighted imaging (SWI) results in a high-contrast image and is sensitive to iron overload (Haller et al., 2021). The nigrosome, a dopaminergic neuron-rich region in the SNc, is the most severely affected region in PD (Kim et al., 2019). Enhanced iron content in the nigrosome leads to the loss of dorsolateral nigral hyperintensity (DNH) on SWI (Pavese and Tai, 2018). Increased iron deposition in the SNc has been linked to the progression of motor impairment and disability in patients with PD (Huang et al., 2020). The absence of DNH on SWI may serve as a new magnetic resonance imaging (MRI) marker for the progression of PD (Pang et al., 2020). Moreover, the loss of the nigrosome on SWI helps distinguish PD from healthy controls, although this approach fails to reliably differentiate PD from atypical parkinsonism (Pavese and Tai, 2018).

Studies have reported interactions of iron and dopamine that are colocalized in dopaminergic neurons of the SNc (Billings et al., 2019). Dopamine replacement treatment, which has neuroprotective effects and prevents age-related iron accumulation in the SNc of mutant mouse models, remains the most effective symptomatic pharmacotherapy for PD (Billings et al., 2019). However, the chelation of iron by levodopa is insufficient to attenuate neuron loss (Billings et al., 2019). In *in vitro* and *in vivo* models, levodopa replacement therapy was shown to be neurotoxic, and the effect of levodopa on iron accumulation was somewhat muted (Billings et al., 2019).

Nevertheless, clinical data have indicated that levodopa either slows the progression of PD or has a long-lasting effect on the symptoms of the disease, but the potential long-term effects of levodopa on PD remain uncertain (Fahn et al., 2004). An in-depth exploration of the prolonged effects of levodopa on iron accumulation in the nigrosome might be helpful in establishing new therapeutic strategies for PD treatment. In this study, a retrospective analysis was used to explore the effects of antiparkinson medications on DNH on SWI, which represents iron accumulation in the nigrosome, in PD patients with different disease durations**.**

## Materials and Methods

### Parkinson’s Disease Patient Information

The neuroimaging data and medical records of 51 patients with PD, nine Parkinson-plus syndrome patients, and 30 healthy subjects were used in this study (different English letters represent the brain images from individual patients). The study was approved by the Ethics Committee of Binzhou Medical University Hospital, and all methods were performed in accordance with the relevant guidelines and regulations. Written informed consent was obtained from all subjects and their legal guardians. Research involving human participants was performed in accordance with the Declaration of Helsinki. Research on patients or healthy volunteers required the supervision of a competent and appropriately qualified physician. This research conformed to generally accepted scientific principles. The physician fully informed the subjects about which aspects of their care were related to the research. Every precaution has been taken to protect the privacy of research subjects and the confidentiality of their personal information. The refusal of a patient to participate in a study or the patient’s decision to withdraw from the study never adversely affected the patient–physician relationship.

**FIGURE 1:**
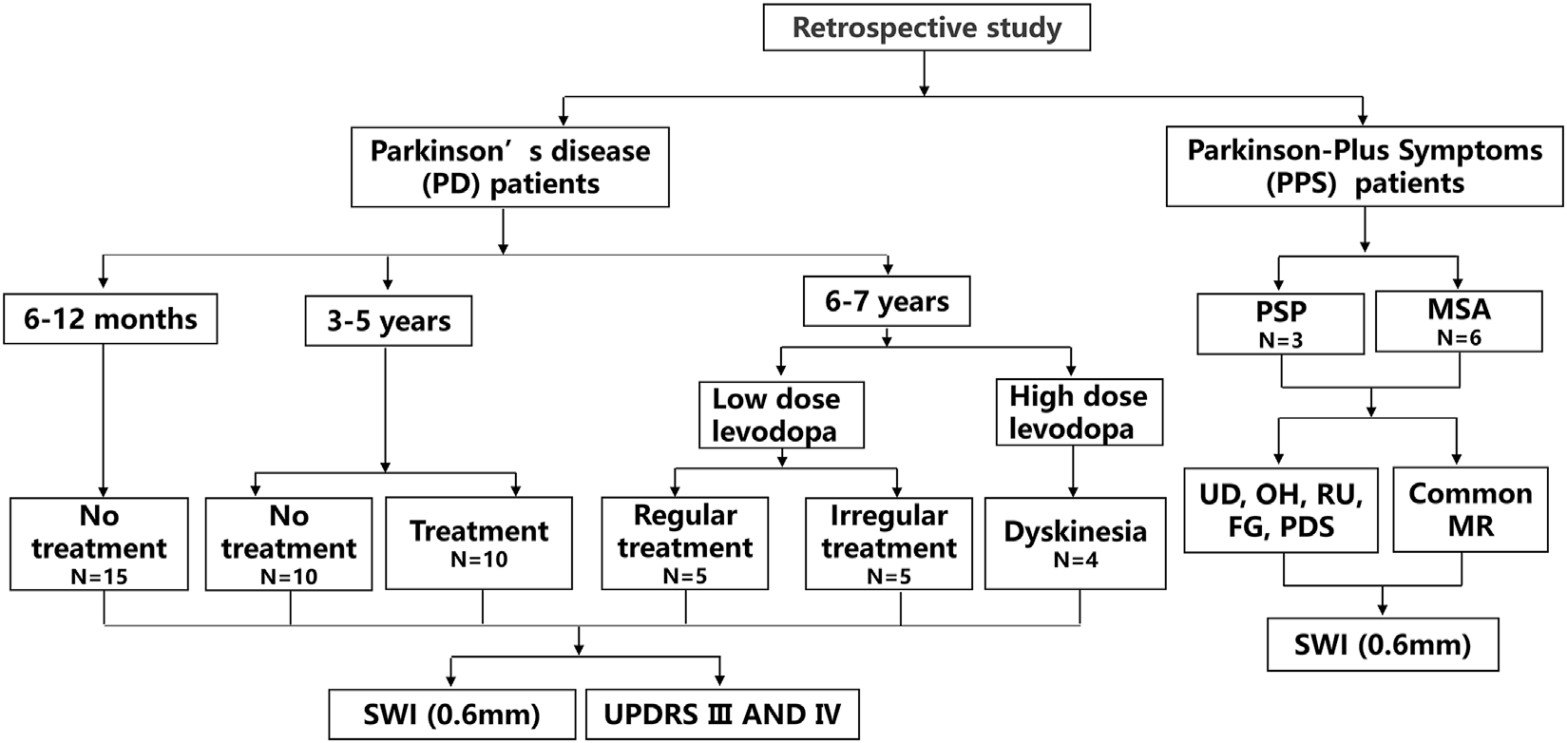
A flowchart of the study is depicted. Progressive supranuclear palsy, (PSP); supranuclear palsy; multiple system atrophy, (MSA); urinary dysfunction, (UD); orthostatic hypotension, (OH); residual urine, (RU); freezing gait, (FG); Parkinsonism, (PDS); and susceptibility-weighted imaging, (SWI).

The no treatment group represented patients who were not administered antiparkinson medications. The treatment group included patients who were administered Madopar (200 mg/50 mg) and selegiline hydrochloride. This regular treatment group included patients who were regularly treated with oral Madopar and selegiline hydrochloride depending on the specialist advice (oral Madopar at fixed time intervals and with a stable oral dosage). Patients with Parkinson-plus syndromes were also treated with Madopar and selegiline hydrochloride in the early stage. A flowchart of the study is shown in Figure 1.

### Visualization of the Nigrosome

Visualization of the nigrosome on SWI with uniform susceptibility was carried out. According to the scores of each side of the midbrain in three cross-sectional planes, bilateral alterations in the nigrosome were evaluated. Nigrosome loss was scored on a 1-point nigrosome visibility scale (1 = unilateral side with normal brightness and present; 0.5 = slightly more difficult to see than normal/reduced size or very difficult to see but identifiable, or possibly parts of the outline are visible but not definitely identifiable; 0 = not identifiable).

### Movement of Parkinson’s Disease Patients was Analyzed Using Unified Parkinson’s Disease Rating Scale III/IVMotor Scores

PD was diagnosed based on the UK Parkinson’s Disease Society Brain Bank diagnostic criteria. PD patients were from Binzhou Medical University Hospital. The severity of PD symptoms was assessed using the Movement Disorder Society revision of the Unified Parkinson’s Disease Rating Scale (MDS-UPDRS) section III (UPDRS III), after the patient had been off levodopa for 24 h (off state). All patients underwent a thorough clinical examination. UPDRS Part IV was used to evaluate the complications of PD, including dyskinesia and dysmyotonia.

### MR Imaging and Image Processing

maging was performed using a 3T MRI device (GE 750w, Amersham, United Kingdom) with a standard body coil for transmitting and a 12-channel head coil for receiving. SWI and T2-weighted sequences were utilized. The volume of the nigrosome was assessed using SWI at 3T with a 0.6-mm slice thickness. Voxel size = 0.75 mm × 0.75 mm × 0.6 mm; field of view 240 mm × 216 mm, TR = 40.3 ms, TE = 24.1 ms, slice: 0.6 mm, Flip Angles: 15, Freq Dir: A/P, Freq. Fov: 24.0, Phase Fov: 0.90, Frequency: 320, Phase: 288, Bandwidth: 41.67. Data were analyzed by three different radiologists.

### Statistical Analysis

The descriptive statistics are presented as the mean ± SEM. Statistical analysis was performed using GraphPad Prism 8.0 **(**GraphPad Software Inc., San Diego, CA, USA**)**. Correlation analysis was used to quantify the degree to which the two variables were related, and *p* < 0.05 was accepted as significant. Pearson correlation analysis was used to assess the relationship between UPDRS Ⅲ and nigrosome scores. Student’s t tests were used to compare the means of two groups. A *p* value of less than 0.05 implies statistical significance.

## Results

### Loss of Dorsolateral Nigral Hyperintensity on Susceptibility Weighted Imaging in Healthy People and Early-Stage Parkinson’s Disease Patients

**FIGURE 2:**
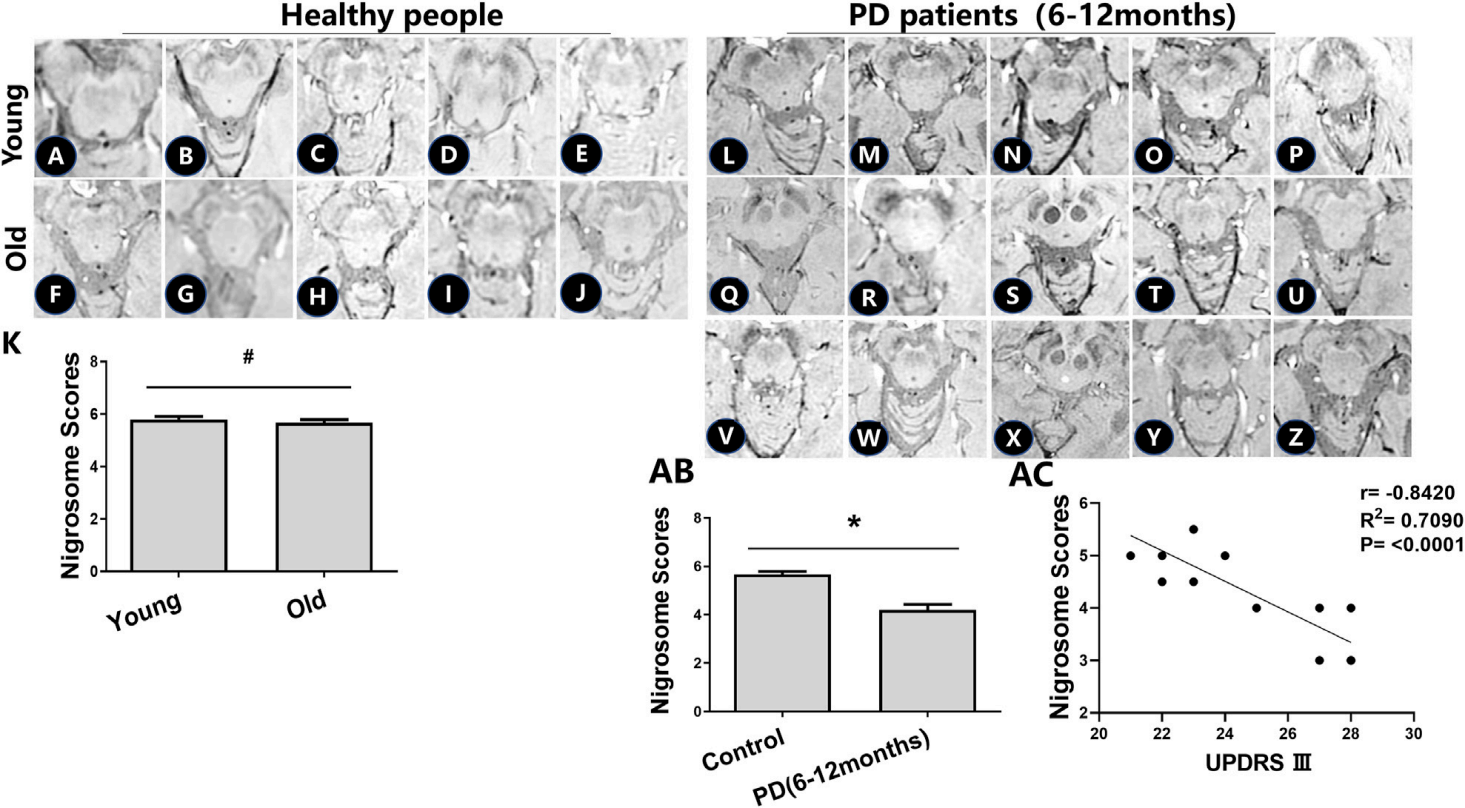
Iron accumulation in the nigrosome of healthy people and PD patients with a 6–12-month disease duration. **(A–J)** Alterations in the nigrosome on SWI in young and healthy older people. **(K)** Mean nigrosome scores were statistically analyzed in young and healthy older people; ^#^ compared with young healthy people, *p* > 0.05. **(L-Z)** Alterations in the nigrosome on SWI in PD patients. **(AB)** Mean nigrosome scores in PD patients were statistically compared with those in healthy older people. Mean nigrosome scores were calculated by utilizing the bilateral nigrosome scores in three axial sections of the brain; * compared with the control group, *p* < 0.05. **(AC)** Correlation analysis was used to evaluate the relationship between UPDRS III and nigrosome scores.

To determine the effects of age-related iron accumulation in the nigrosome, young (30–50 years) and old (51–80 years) healthy people without brain disease were chosen. DNH was observed in healthy people. In healthy control subjects, there was no significant difference in the nigrosome scores between young and old healthy people (Figures 2A–J).

There had been no drug treatment for PD patients with a 6–12-month disease duration who were seeking treatment for the first time. Patient information, including sex, UPDRS III scores, Hoehn and Yahr scale scores, and nigrosome scores, is recorded in Table 1. The alteration of substantia nigra hyperintensity was recorded in SWI, and nigrosome scores were calculated using the 1-point nigrosome visibility scale (Figures 2L–Z). According to the modified Hoehn and Yahr scale, the patients were classified as stage 1. The UPDRS III scores ranged from 20 to 30. The bilateral asymmetric loss of signal hyperintensity of the nigrosome was shown on SWI. There was a significant difference in the nigrosome scores between old healthy people and PD patients (Figure 2AB, *p* < 0.05). Correlation analysis indicated that the UPDRS III scores negatively correlated with nigrosome scores in the patients with a 6–12-month disease duration (*r* = −0.8420, Figure 2AC).

**TABLE 1:** The information of PD patients who had no drug treatment with a 6–12 month disease duration.

Patients	Gender	Age (Y)	UPDRS III	H&Y	Nigrosme scores
L	F	56	28	1	3
M	F	54	27	1	3
N	N	59	23	1	5.5
O	F	61	28	1	3
P	F	54	23	1	4.5
Q	M	52	24	1	5
R	F	56	28	1	3
S	M	61	27	1	4
T	F	62	28	1	4
U	F	59	27	1	4
V	F	54	22	1	4.5
W	F	57	21	1	5
X	F	58	25	1	4
Y	F	61	22	1	5
Z	F	51	23	1	5.5

### Effects of Levodopa Therapy on Dorsolateral Nigral Hyperintensity

**FIGURE 3:**
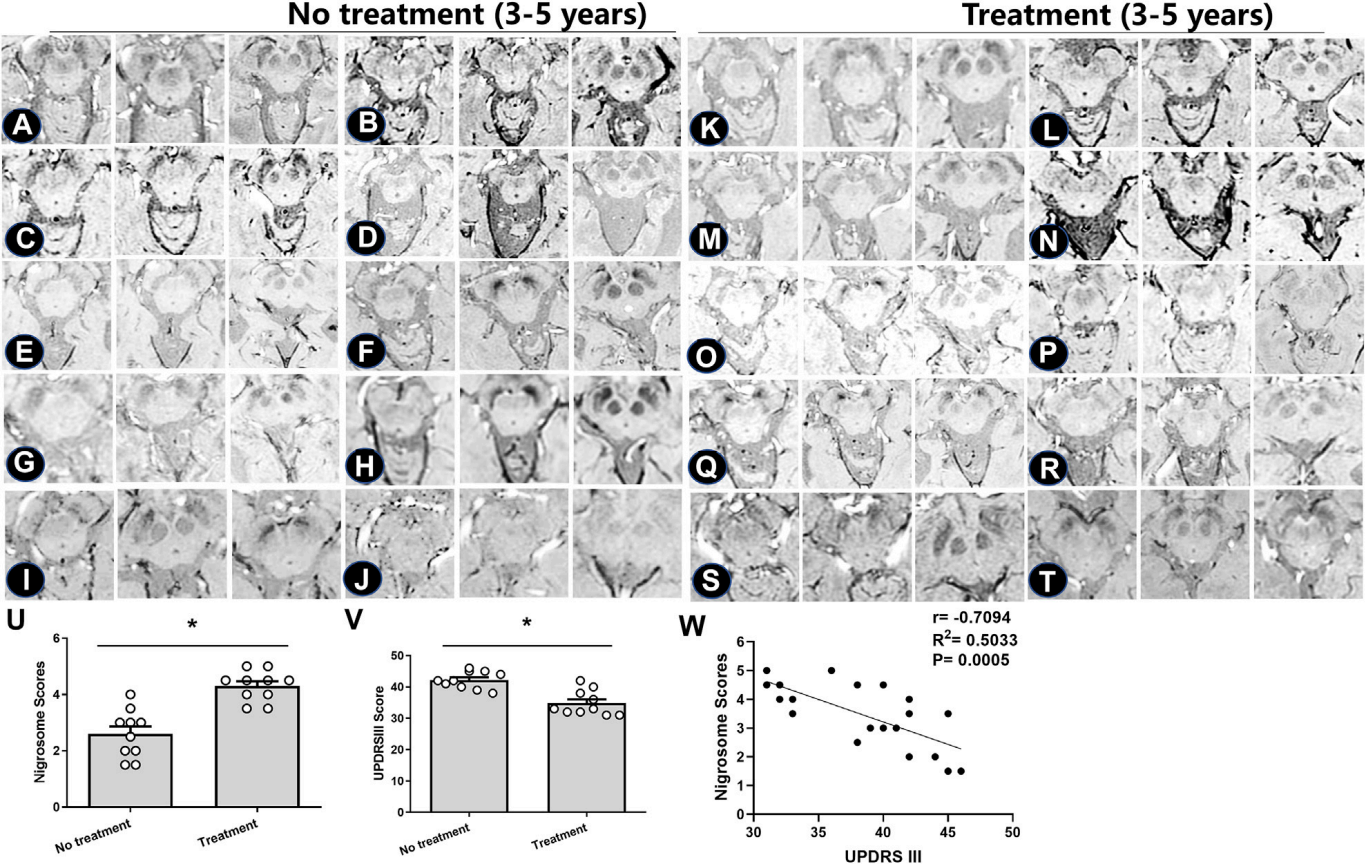
Iron deposition in the substantia nigra pars compacta in individual patients treated with different antiparkinson medications. **(A–T)** SWI images of 20 PD patients. Based on levodopa treatment, the absence of the nigrosome was evaluated and detected. **(U,V)** Nigrosome scores and UPDRS Ⅲ scores were statistically analyzed in PD patients; * compared with the untreated group, *p* < 0.05. Mean scores were calculated based on the scores in three axial sections of the brain. **(W)** Correlation analysis was used to evaluate the relationship between UPDRS Ⅲ scores and nigrosome scores.

For those with a 3–5-year duration of PD, the alterations in the nigrosome on SWI were investigated depending on the different treatments (Figures 3A–T). Patient information is recorded in Table 2. There was no significant difference in the average age of patients between the treatment and no treatment groups (Table 2). The loss of the nigrosome was statistically analyzed according to the 1-point nigrosome visibility scale on high-resolution 3T-SWI. Compared with those in untreated patients, the nigrosome scores were increased owing to early treatment with antiparkinson drugs (*p* < 0.05) (Figure 3U). There is a significant difference in UPDRS Ⅲ scores between the treatment and no treatment groups (Figure 3V). Correlation analysis indicated that the UPDRS Ⅲ scores negatively correlated with nigrosome scores (*r* = −0.7094, Figure 3W).

**TABLE 2:** The information of PD patients with a 3–5 year duration between the treatment and no treatment groups.

Patients		Age(Y)	Gender	H&Y	Average age (mean ± SD)
No treatment	A	70	M	3	65.4 ± 6.7
	B	59	M	3	
	C	63	F	3	
	D	64	M	3	
	E	58	F	3	
	F	77	F	3	
	G	72	F	3	
	H	68	M	3	
	I	69	M	3	
	J	54	F	3	
Treatment	K	74	M	3	63.9 ± 8.1^#^
	L	55	F	3	
	M	56	M	3	
	N	58	M	3	
	O	63	F	3	
	P	73	F	3	
	Q	65	M	3	
	R	76	M	3	
	S	67	F	3	
	T	52	F	3	

^#^Compared with no treatment group, *p* > 0.05.

**FIGURE 4:**
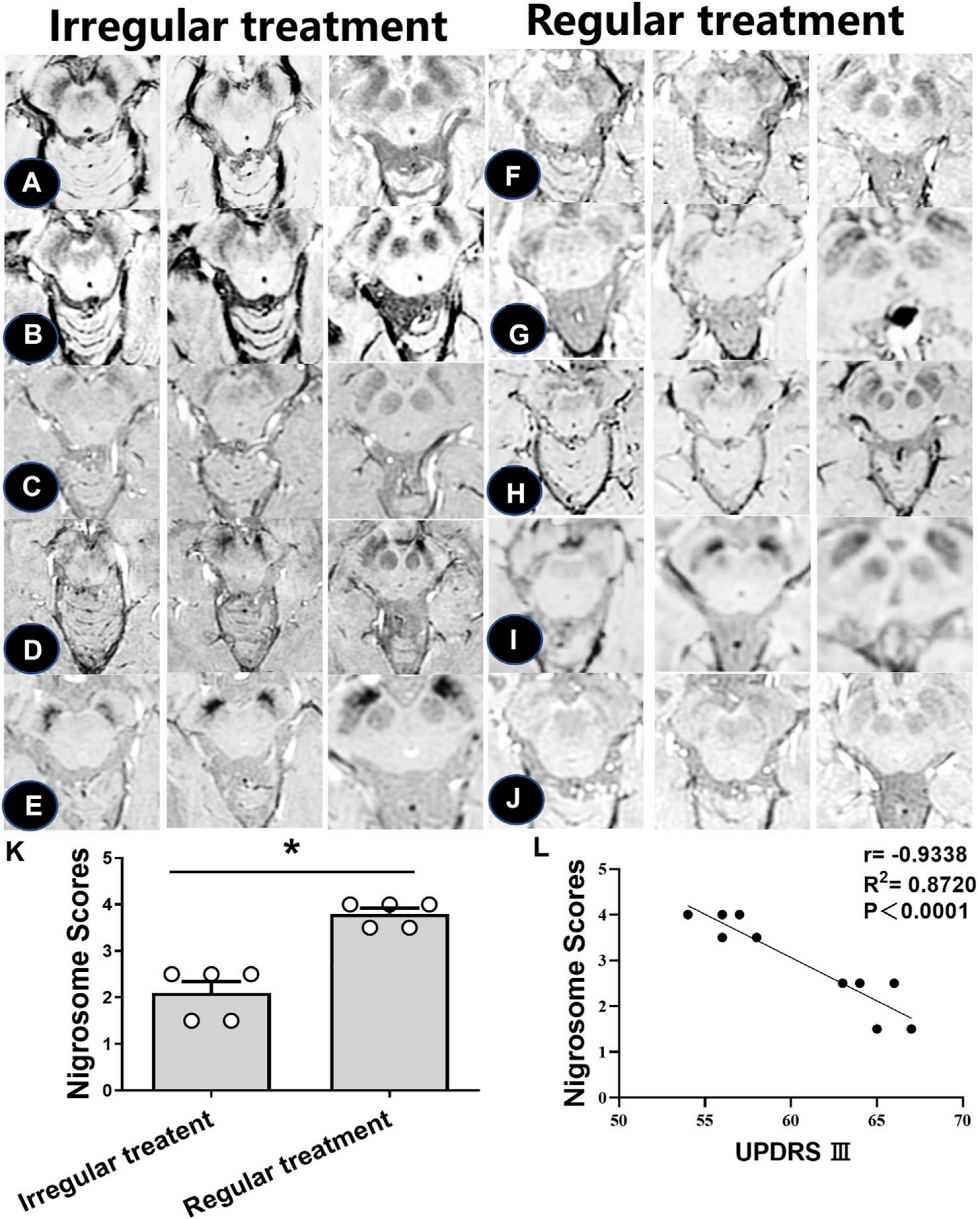
Iron accumulation in the nigrosome with regular and irregular treatments. **(A–J)**. SWI images of 10 PD patients receiving irregular or regular levodopa treatment. **(K)** Mean scores of the nigrosome was increased with regular treatment compared with irregular treatment; * compared with irregular treatment, *p* < 0.05. **(L)** Correlation analysis was used to reveal the relationship between UPDRS Ⅲ scores and nigrosome scores.

Subsequent analyses were performed to investigate the loss of DNH in patients with irregular and regular treatment. As shown in Table 3, the nigrosome scores were increased from 2.1 ± 0.5 to 3.8 ± 0.2 in those with regular oral administration of Madopar and selegiline hydrochloride compared with those with irregular treatment (*p* < 0.05) (Figures 4A–K). The motor symptoms of PD were ameliorated according to UPDRS Ⅲ scores from 65 ± 1.4 to 56.2 ± 1.3 owing to regular treatment. UPDRS Ⅲ scores were negatively correlated with nigrosome scores (*r* = −0.9338) (Figure 4L).

**TABLE 3:** The information of patients with irregular and regular treatment.

Patients		Age(Y)	Gender	Duration (Y)	H&Y	UPDRS III (mean ± SD)	Nigrosome score (mean ± SD)
Irregular treatment	A	65	F	7	3	65 **±** 1.4	2.1 **±** 0.5
	B	68	F	6	3		
	C	66	F	6	3		
	D	72	M	6	3		
	E	75	M	7	3		
Regular treatment	F	63	M	6	3	56.2 **±** 1.3	3.8 **±** 0.2
	G	72	M	7	3		
	H	71	M	6	3		
	I	73	F	7	3		
	J	66	M	6	3		

**FIGURE 5:**
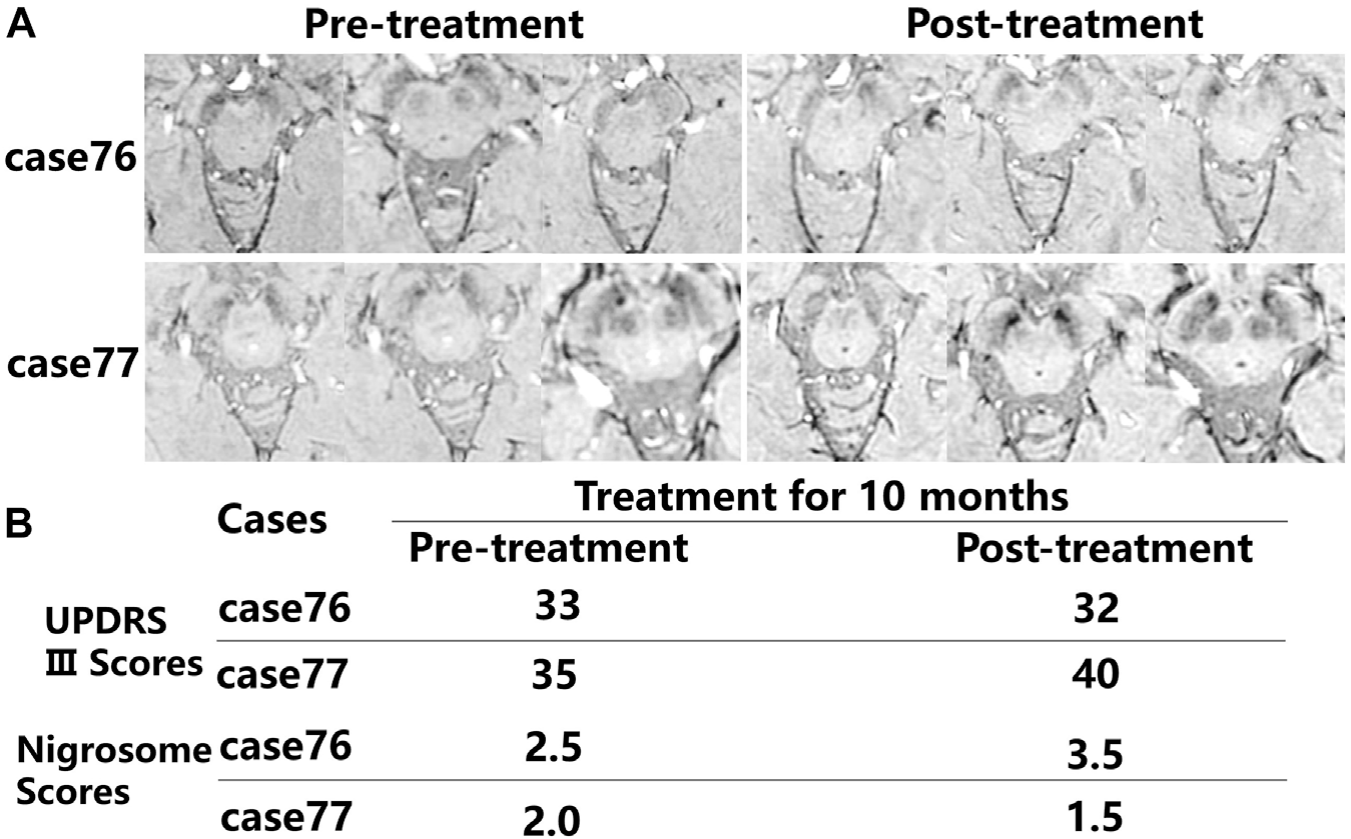
Comparison in the alterations in iron accumulation in the nigrosome in the pretreatment and posttreatment groups. **(A)** The alterations in iron deposition in the nigrosome were evaluated in two of the patients after treatment for 10 months. **(B)** Nigrosome scores and UPDRS Ⅲ scores were observed after receiving pharmacological treatment.

After administration of levodopa for 10 months, the alterations in iron deposition in the nigrosome were different in two of the patients (Figures 5A,B). For patient case 76 receiving regular oral Madopar and selegiline hydrochloride, increased nigrosome scores and unchanged UPDRS III scores were detected. For patient case 77, compared with pretreatment scores, decreased nigrosome scores and increased UPDRS III scores were observed owing to irregular treatment.

### Loss of Dorsolateral Nigral Hyperintensity was Prevented in Patients with a High Dose of Levodopa

**FIGURE 6:**
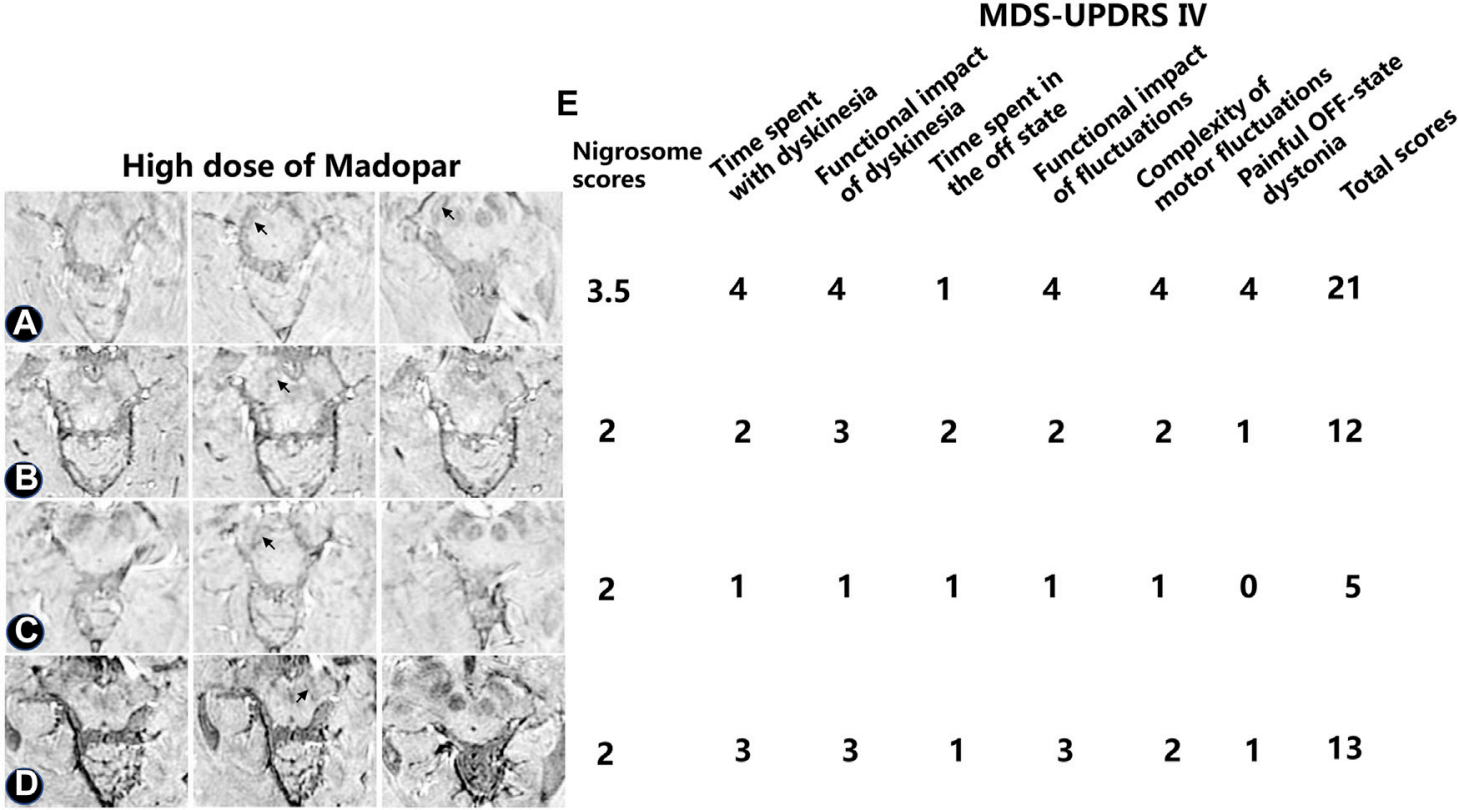
Alterations in iron aggregation in the substantia nigra in four PD patients treated with high-dose levodopa with peak-dose dyskinesia or biphasic dyskinesia. The nigrosome was visible as a bright area **(A–D)** The nigrosome was visible (black arrow) as a bright area. **(E)** The dyskinesia was evaluated by utilizing the UPDRS Ⅳ.

UPDRS IV scores were used to evaluate the motor complications induced by levodopa (Figure 6). In one patient treated with high-dose levodopa for 7–8 years, there was no apparent loss of DNH on SWI, as shown in Figure 6A (black arrow). Four of the PD patients had peak-dose dyskinesia or biphasic dyskinesia. A greater severity of dyskinesia with a high UPDRS IV score and higher nigrosome scores was revealed in patient A than in patients B–D (Figure 6, black arrow).

### The Early Levodopa Treatment for the Alteration of Iron Deposition in Parkinson-Plus Symptoms

**FIGURE 7:**
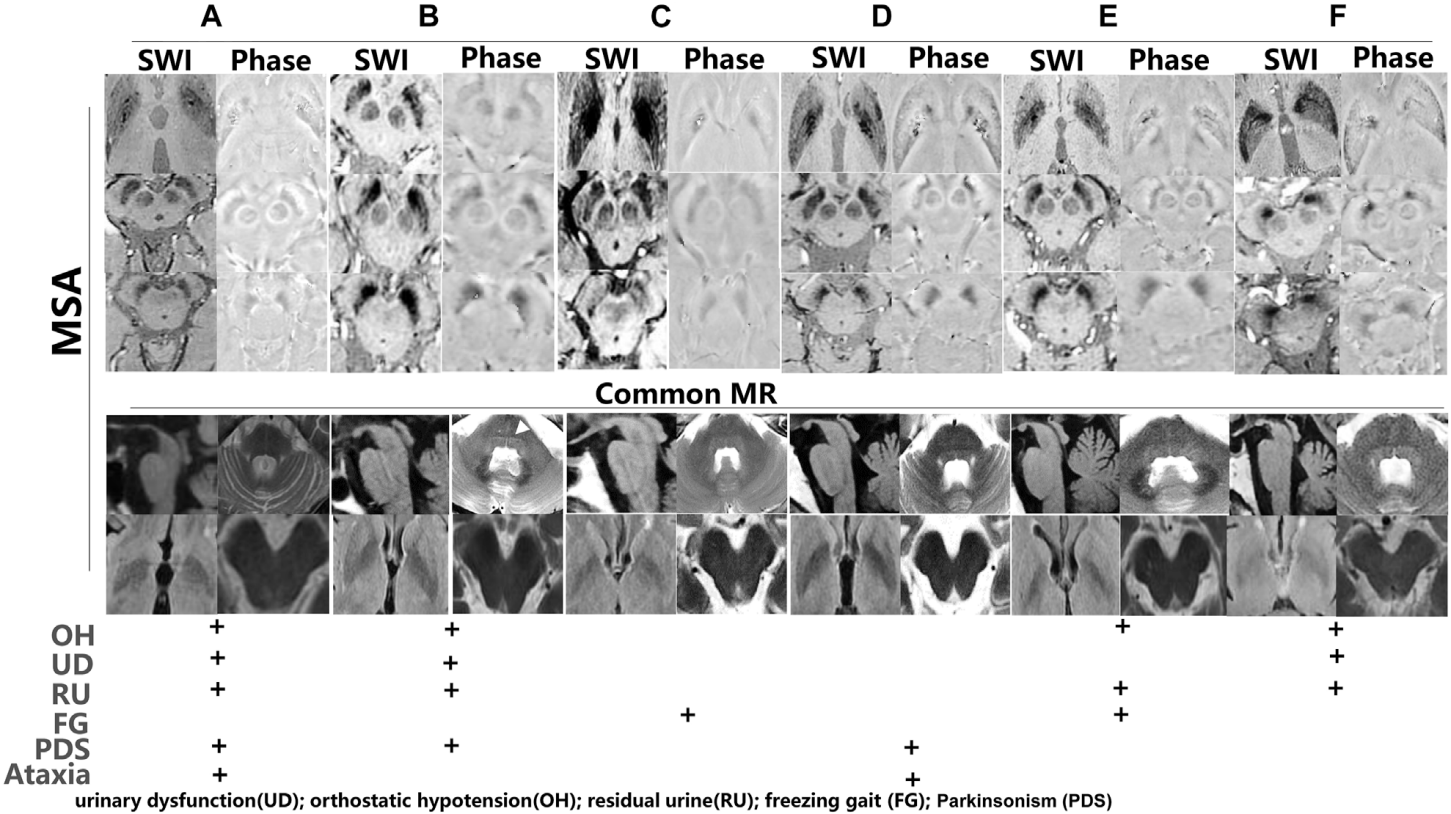
Iron accumulation depicted in multiple system atrophy (MSA). **(A–F)** Iron accumulation in the substantia nigra and corpus striatum of MSA patients (B, white arrow: hot cross bun sign). Phase imaging of SWI was used to differentiate the presence of calcium from iron. Common brain MR and symptoms such as urinary dysfunction (UD), orthostatic hypotension (OH), residual urine (RU), freezing gait (FG), and parkinsonism (PDS) were used to diagnose MSA.

**FIGURE 8:**
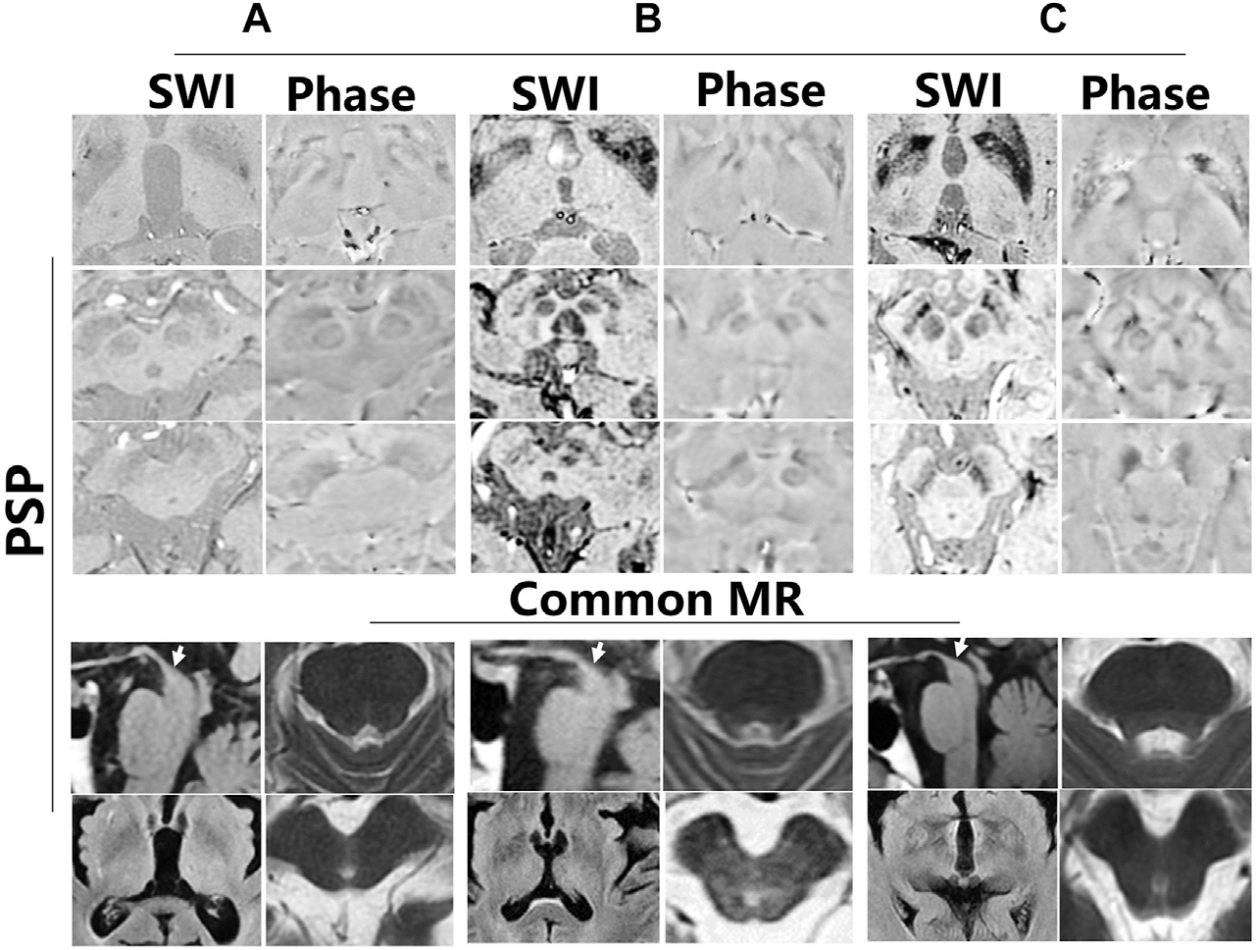
Iron deposition in the substantia nigra and corpus striatum of progressive supranuclear palsy (PSP). **(A–C)** Different patients with PSP. There is a hummingbird sign on sagittal T1WI of the brain in these patients (white arrow).

To further investigate iron accumulation in those with Parkinson-plus syndrome, alterations in signal hyperintensity in the nigrosome in nine patients were examined. As Figure 7 indicates, patients A–F were diagnosed with multiple system atrophy (MSA) depending on clinical evidence of urinary dysfunction (UD), orthostatic hypotension (OH), residual urine (RU), freezing gait (FG), parkinsonism (PDS), and ataxia and on neuroimaging evidence including a hot cross bun sign in the pons on a 3T MRI (Figure 7B, white arrow). As Figure 7 shows, hypointensity increased in the substantia nigra and corpus striatum of patients on SWI. Figures 8A–C indicates hypointensity in the corpus striatum of patients diagnosed with probable progressive supranuclear palsy (PSP). The hummingbird sign was seen on sagittal T1WI of the brain in patients A and B (Figures 8A,B, white arrow).

## Discussion

In this study, the retrospective data suggested that the clinical symptoms of PD with no drug treatment quickly progressed and resulted in physiological deformities. Nevertheless, early and regular treatment with levodopa decreased UPDRS III motor scores and the loss of DNH. The rational administration of levodopa not only delayed the progression of PD but also affected iron deposition in the nigrosome.

The imaging of nigrosome loss has been recognized as a biomarker of the pathogenesis of PD (Sung et al., 2021). Additional evidence suggested that poor visualization of the nigrosome in PD provides diagnostic evidence of the disease (Billings et al., 2019). The loss of the nigrosome was significantly associated with higher motor asymmetry on the contralateral side in subjects (Billings et al., 2019). In our study, all PD patients had different degrees of nigrosome volume loss compared with healthy people. Although some studies have suggested that the nigrosome had the typical “swallow tail” appearance (Lee et al., 2021), a greater volume of the nigrosome was observed in the SNc by using SWI with different susceptibilities. Moreover, SWI measures iron accumulation with a semiquantitative pattern and can be used to track iron accumulation in PD (Weinreb et al., 2013; Takahashi et al., 2018). In our study, SWI at 3T was adequate for showing the alteration of iron accumulation in the nigrosome.

Some studies have indicated that in the SNc, metabolic disturbances associated with levodopa are related to iron accumulation (Billings et al., 2019). Iron is essential for many biological functions, including neurotransmitter synthesis, where the metal is a cofactor of tyrosine, which converts tyrosine to dopamine (Zecca et al., 2008). In previous studies, only dopamine but not tyrosine or norepinephrine interfered with cellular iron homeostasis and promoted cellular iron accumulation (Dichtl et al., 2018). Furthermore, increases in intracellular iron with dopamine therapy result in the production of reactive oxygen species (ROS) and neurodegeneration (Dichtl et al., 2018). Targeting iron and dopamine interactions may be an effective way to modify neuronal vulnerability and thus slow neuronal loss (Hare and Double, 2016). In another study, levodopa was shown to be neuroprotective and to prevent age-related iron accumulation in the substantia nigra (Beard, 2001; Fahn et al., 2004). Our retrospective study indicated that early and regular treatment with levodopa prevented the loss of DNH and alleviated the symptoms of PD. A recent study suggested that the nigral iron deposition was associated with levodopa-induced dyskinesia (Song et al., 2021). However, in our study, decreased loss of DNH was observed in one patient with more severe dyskinesia.

**FIGURE 9:**
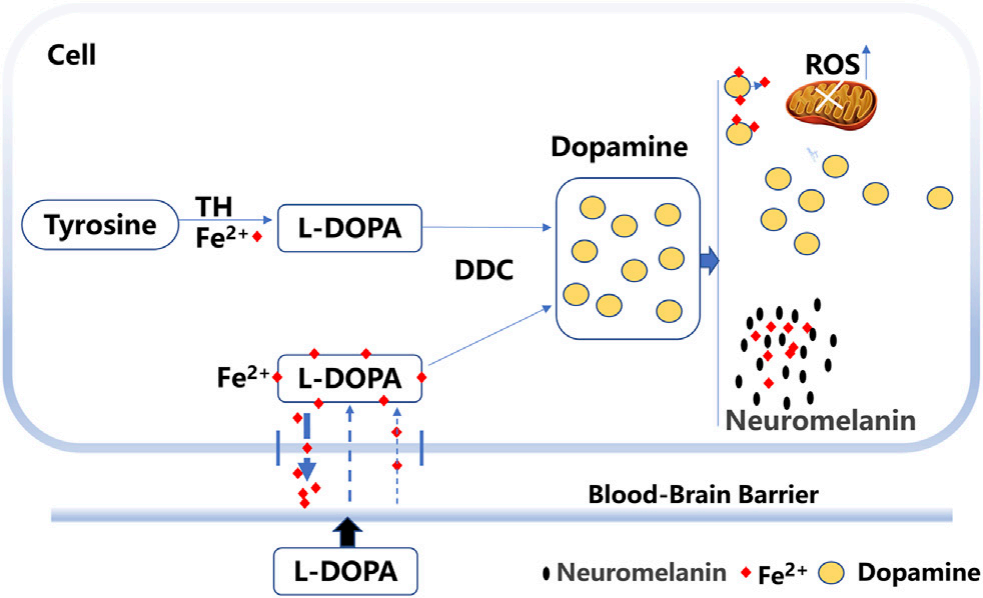
Probable mechanism for the effects of levodopa on iron deposition. Levodopa (L-DOPA) can cross the blood barrier and form a stable complex through binding to iron. The levodopa–iron complex facilitates iron transport out of cells. Excess dopamine is converted to neuromelanin and enhances the binding of neuromelanin to iron. Levodopa treatment also induced the production of reactive oxygen species (ROS).

Studies have indicated that levodopa binding to iron forms a stable complex (Alhassen et al., 2022). The levodopa molecule is known to contain a domain with iron-chelating properties or the ability to prevent iron deposition and the toxic effects of extra free iron (Billings et al., 2019; Alhassen et al., 2022). Iron transport is regulated by the hepcidin–ferroportin system to control iron release into the plasma (Wu et al., 2021). Hepcidin expression is principally regulated by plasma iron in a feedback loop (Zucca et al., 2017). In our study, excess extracellular levodopa binds iron, which reduces the plasma iron level and regulates hepcidin expression, which might subsequently facilitate iron transport out of cells and decrease iron deposition (Figure 9).

In addition, excess dopamine is converted to neuromelanin, and iron binds to neuromelanin in the human SNc (Zucca et al., 2017). Neuromelanin was shown to be markedly decreased in the SNc of PD patients, and neuromelanin represented the major iron storage capacity in substantia nigra neurons (Zecca et al., 2001; Nagatsu et al., 2022). Neuromelanin serves to trap iron, which results in a high load of iron in the brain (Zucca et al., 2017). After binding to neuromelanin, iron changes its location or state (Zucca et al., 2017). The accumulation of neuromelanin increases the free Fe^2+^ form in the SNc of patients with PD (Faucheux et al., 2003). Whether neuromelanin enhances the binding activity to iron with long-term levodopa treatment remains to be elucidated. Thus, iron bound to neuromelanin might be another mechanism for decreased iron levels (Figure 9).

Previous evidence has suggested that MSA and PSP patients have obvious iron accumulation in different brain regions (Lee and Lee, 2019). Histopathological studies in MSA patients revealed that iron deposition in the putamen was a hallmark of the disease (Kaindlstorfer et al., 2018). Similarly, our study showed the enhancement of hypointensity in the substantia nigra and corpus striatum on SWI in MSA patients. The PSP group had significantly increased iron content in the globus pallidus and caudate nucleus (Lee et al., 2013). In our study, increased hypointensity in the globus pallidus and putamen was observed in PSP patients.

In our study, by comparing the effects of different treatments on the loss of DNH, the view that proper treatment with levodopa prevented iron deposition in the nigrosome was validated. Nigrosome scores were calculated objectively using the 1-point nigrosome visibility scale. However, a small sample size can also lead to the introduction of bias and cannot exclude the influence of individual differences in iron content in the nigrosome. The genuine effects of levodopa treatment on iron deposition need more clinical evidence. In future studies, we will investigate alterations in iron accumulation in the nigrosome between patients from pretreatment to posttreatment. In summary, early rational pharmacotherapy might prevent iron accumulation in the nigrosome and ameliorate motor symptoms.

## Data Availability

The original contributions presented in the study are included in the article/Supplementary Material, and further inquiries can be directed to the corresponding authors.
