# Supplementary figures and images for: Modified Iron Deposition in Nigrosomes by Pharmacotherapy for the Management of Parkinson’s Disease
Source: Front Mol Biosci. 2022 Jul 7;9:908298. doi: 10.3389/fmolb.2022.908298 (PMC9301007; doi:10.3389/fmolb.2022.908298)

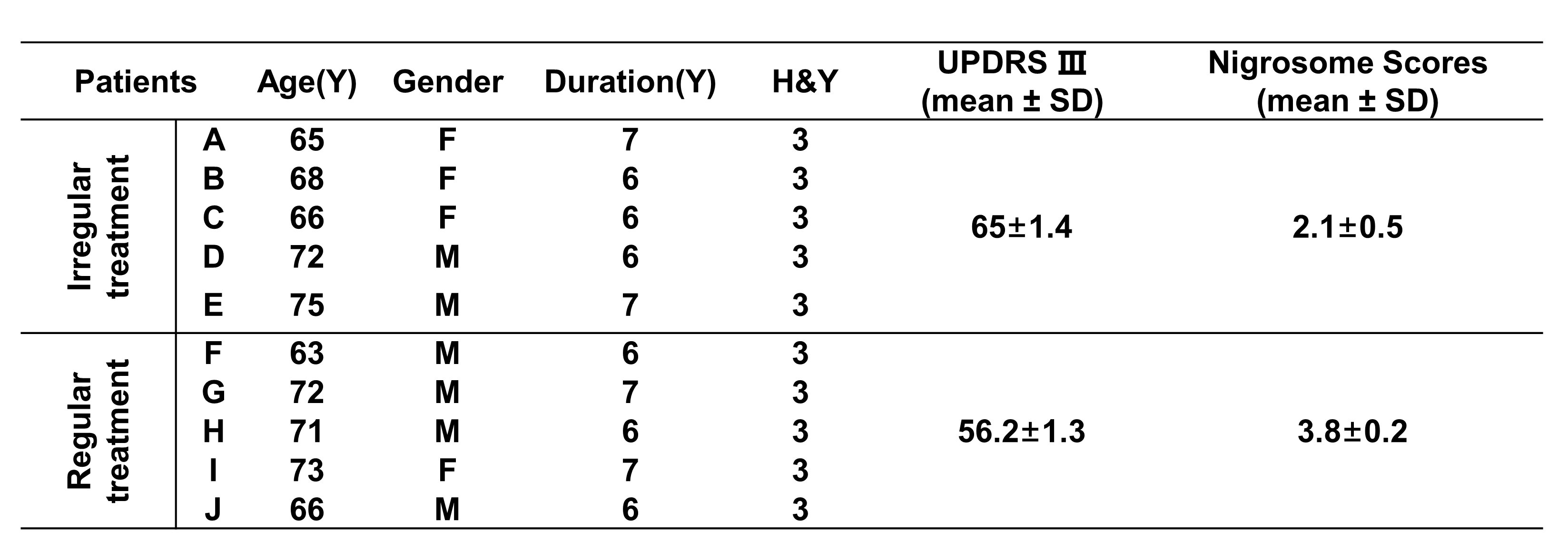

Supplement: Supplementary file 1 [file Image3.JPEG]

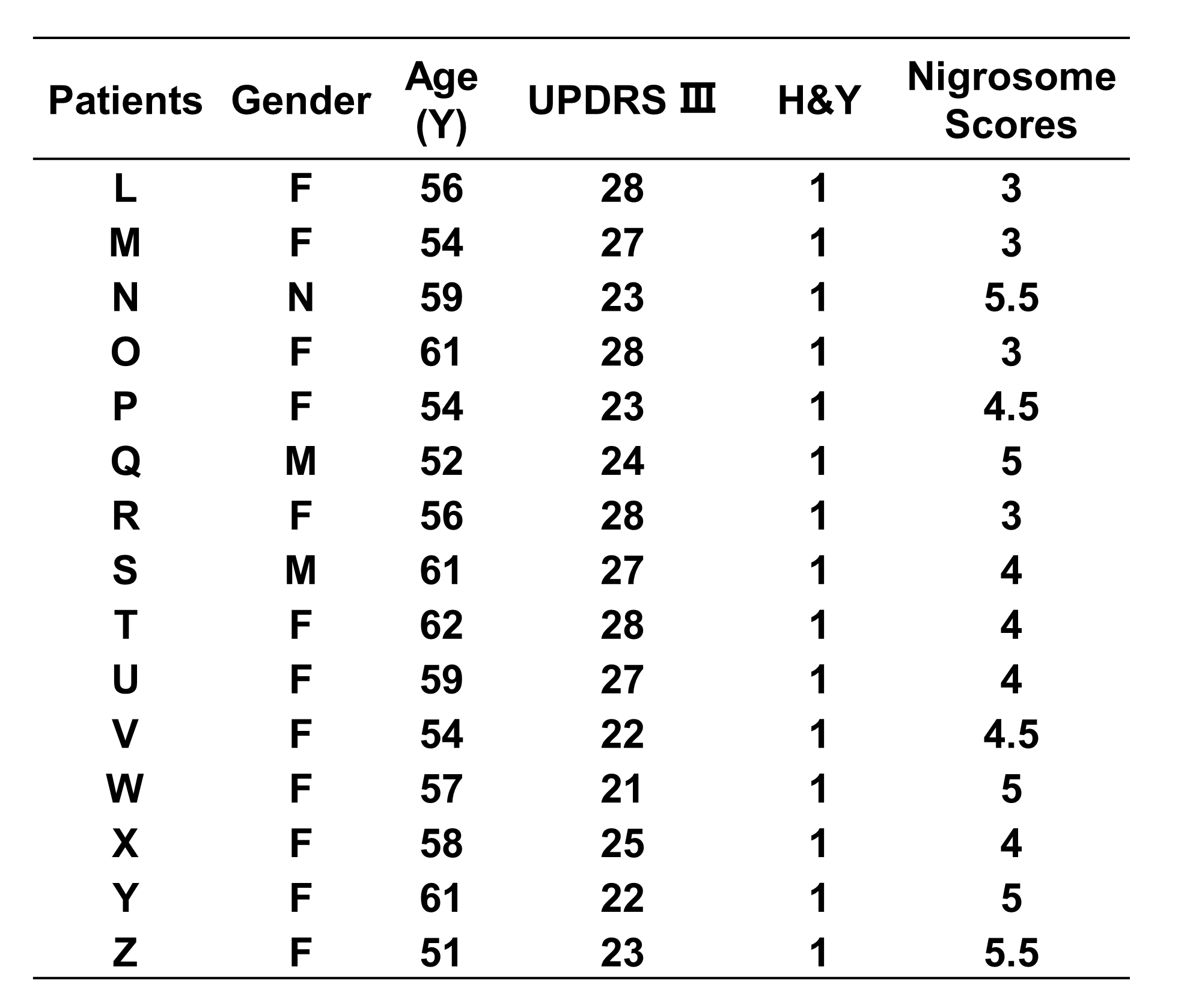

Supplement: Supplementary file 2 [file Image1.JPEG]

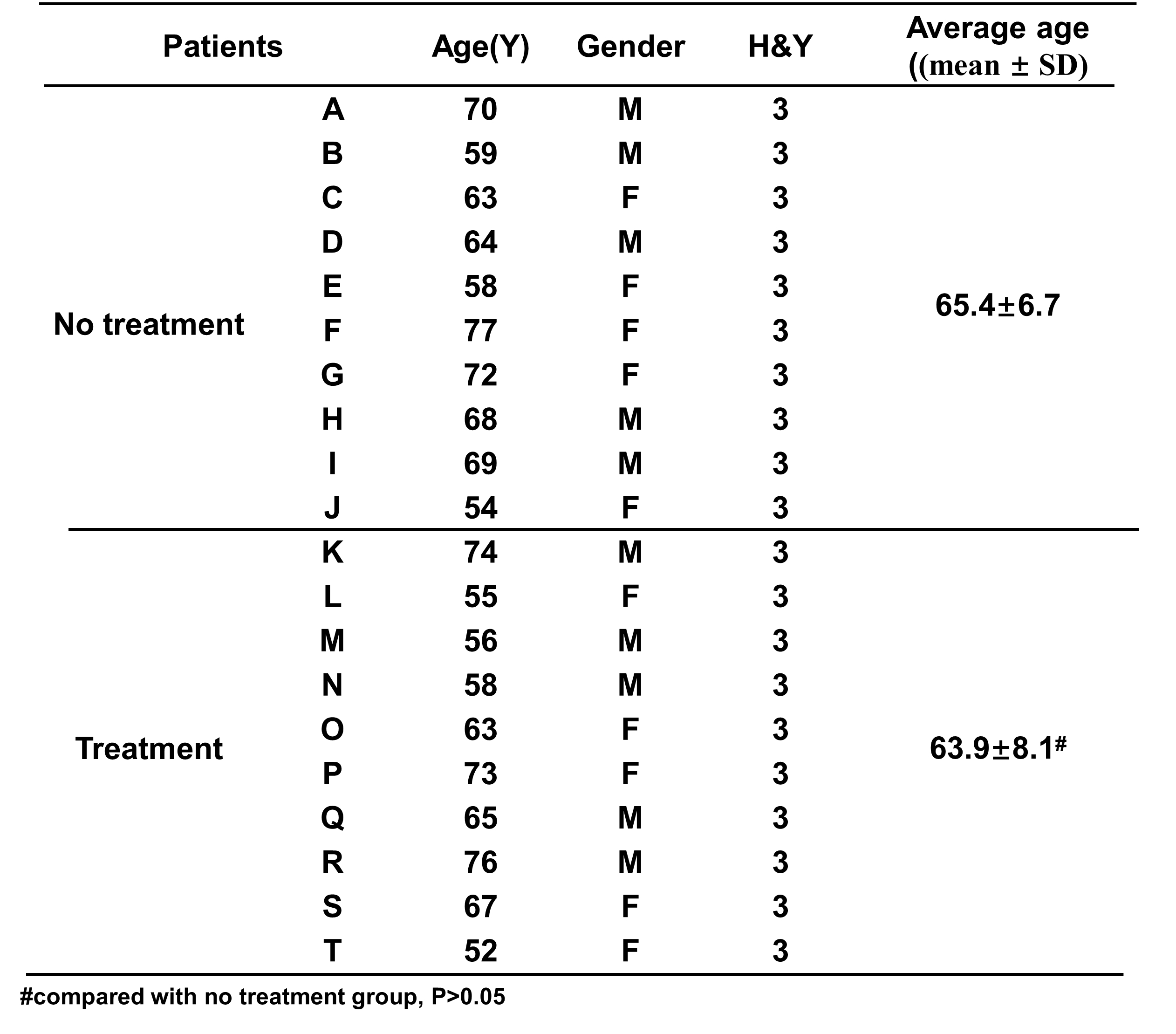

Supplement: Supplementary file 3 [file Image2.JPEG]
